# ﻿New insights into the phylogenetic relationships of Japanese knotweed (*Reynoutriajaponica*) and allied taxa in subtribe Reynoutriinae (Polygonaceae)

**DOI:** 10.3897/phytokeys.220.96922

**Published:** 2023-02-27

**Authors:** Stuart D. Desjardins, John P. Bailey, Baowei Zhang, Kai Zhao, Trude Schwarzacher

**Affiliations:** 1 Department of Genetics and Genome Biology, University of Leicester, Leicester (Leicestershire), UK University of Leicester Leicester United Kingdom; 2 School of Life Sciences, Anhui University, Hefei (Anhui), China Anhui University Hefei China; 3 The National Engineering Laboratory of Crop Stress Resistance Breeding, Anhui Agricultural University, Hefei (Anhui), China Anhui Agricultural University Hefei China

**Keywords:** *Fallopia*, invasive aliens, *Muehlenbeckia*, phylogeny, polyploidy

## Abstract

Japanese knotweed (*Reynoutriajaponica*) is native to East Asia, but has been introduced to the West where it is a noxious invasive weed. Taxonomically, Japanese knotweed is placed within subtribe Reynoutriinae (Polygonaceae), which also contains the austral genus *Muehlenbeckia* (incl. *Homalocladium*) and north temperate *Fallopia*. In the current study, we conducted a phylogenetic analysis using sequence data from six markers, two nuclear (*LEAFYi2*, ITS) and four plastid (*matK*, *rbcL*, *rps16-trnK* and *trnL-trnF*) to further resolve the evolutionary relationships within this group, using the widest sampling of in-group taxa to date. The results of this analysis confirmed that subtribe Reynoutriinae is a monophyletic group, characterised by the presence of extra-floral, nectariferous glands at the base of leaf petioles. Within the subtribe, four main clades were identified: *Reynoutria*, Fallopiasect.Parogonum, *Fallopia* s.s. (including *Fallopia* sects. *Fallopia* and *Sarmentosae*) and *Muehlenbeckia*. The *Fallopia* s.s. and *Muehlenbeckia* clades are sister to one another, while the Fallopiasect.Parogonum clade is immediately basal to them and *Reynoutria* basal to all three. *Fallopia*, as currently circumscribed, is paraphyletic as *Muehlenbeckia* is nested within it. To resolve this, we propose that species of Fallopiasect.Parogonum should be treated as a new genus, *Parogonum* (Haraldson) Desjardins & J.P.Bailey, gen. et stat. nov. Within *Reynoutria*, the allied specific and infraspecific taxa that fall under the name Japanese knotweed s.l. form a monophyletic group and their taxonomic status is discussed.

## ﻿Introduction

Japanese knotweed *sensu lato* is a group of large rhizomatous herbs in the genus *Reynoutria* Houtt. (Ohwi 1965; Anjen and Park 2003b). They are native to East Asia, but have been introduced to the West where they are invasive and persistent weeds (Bailey and Conolly 2000). There are two main species of knotweed: Japanese knotweed (*R.japonica* Houtt.) and giant knotweed (*R.sachalinensis* (F.Schmidt) Nakai) (Ohwi 1965; Anjen and Park 2003b). *Reynoutriajaponica* can also be further recognised as a number of allied specific or infraspecific taxa, most of which are endemic to East Asia, but two are found outside of the native range, a tall lowland form, var.japonica (*R.japonica* s.s.) and a dwarf montane form, var.compacta (Hook.f.) Buchheim (= *Reynoutriacompacta* (Hook.f.) Nakai) (Bailey 2003). Introduced knotweeds show greatly reduced genetic diversity compared to those in the native range, due to strong founder effects (Hollingsworth and Bailey 2000; Pashley 2003; Desjardins et al. 2022). This is most pronounced in R.japonicavar.japonica, which occurs throughout Europe as a single female clone, that spreads by massive clonal reproduction and only produces seed through hybridisation with related taxa (Hollingsworth and Bailey 2000; Pashley 2003; Mandák et al. 2005).

Within the Polygonaceae, Japanese knotweed s.l. is placed in subtribe Reynoutriinae (Galasso et al. 2009), which is characterised by two putative synapomorphies: extra-floral nectaries at the base of leaf petioles (Salisbury 1909; Brandbyge 1992; Schuster et al. 2011b) and *Tiniaria*-type pollen (Hedberg 1946; Bailey 1989; Brandbyge 1992). In addition to the East Asian knotweeds (*Reynoutria*), the subtribe contains the austral genus *Muehlenbeckia* Meisn. (including *Homalocladium* (F.Muell.) L.H.Bailey) and the north-temperate genus *Fallopia* Adans.; all of which are segregates of *Polygonum* L. s.l. (Schuster et al. 2011b).

*Fallopia* and *Reynoutria* have been treated as a single entity ever since Meisner (1856) placed them together in Polygonumsect.Tiniaria Meisn. and, thereafter, by Hedberg (1946) under *Tiniaria* (Meisn.) Rchb., by Shinners (1967) under *Reynoutria*, by Ronse Decraene and Akeroyd (1988) under *Fallopia* and by Galasso et al. (2009) as separate genera under subtribe Reynoutriinae. *Muehlenbeckia*, however, has traditionally been considered distinct from *Fallopia* and *Reynoutria*, primarily on the basis of its succulent mature perianth and southern biogeographical distribution. Meisner (1840, 1856) instigated this by segregating *Muehlenbeckia* from *Polygonum* s.l. and the rest of the tribe Polygoneae and placing it in tribe Coccolobeae alongside *Coccoloba* P.Browne, which also has inflated tepals in fruit. This classification persisted until relatively recently, being adopted as late as Brandbyge (1993), and was not re-examined until the application of molecular techniques (Cuénoud et al. 2002; Lamb Frye and Kron 2003). However, earlier workers, such as Jaretzky (1925) and Edman (1929), had suggested that *Muehlenbeckia* may be derived from Polygonumsect.Pleuropterus (Turcz.) Benth. & Hook.f. (= *Reynoutria*), due to similarities in secondary chemistry and endosperm morphology. Furthermore, Haraldson (1978) suggested that the closest connection of *Fallopia* and *Reynoutria* was probably with *Muehlenbeckia*, amongst other genera, as a number of morphological traits, such as fimbriate stigmas and twining habit, are found within both groups. To indicate this relationship, she transferred *Fallopia* and *Reynoutria* into the Coccolobeae to be alongside *Muehlenbeckia*. A summary of the historical treatments of *Fallopia*, *Reynoutria* and *Muehlenbeckia* is presented in Table 1.

**Table 1.** Taxonomic treatment of *Fallopia*, *Muehlenbeckia*, *Parogonum* and *Reynoutria* in previous classifications.

	*Fallopia*	*Muehlenbeckia*	*Parogonum*	*Reynoutria*
**Meisner (1856)**	Polygonumsect.Tiniaria	*Muehlenbeckia*	Polygonumsect.Tiniaria	Polygonumsect.Tiniaria
**Bentham and Hooker (1880)**	Polygonumsect.Tiniaria	*Muehlenbeckia*	n/a	Polygonumsect.Pleuropterus
**Nakai (1926)**	*Bilderdykia*	n/a	n/a	*Reynoutria*
**Hedberg (1946)**	*Tiniaria*	n/a	*Tiniaria*	*Tiniaria*
**Webb and Chater (1963)**	*Bilderdykia*	n/a	n/a	*Reynoutria*
**Shinners (1967)**	*Reynoutria*	n/a	*Reynoutria*	*Reynoutria*
**Holub (1970)**	*Fallopia*	n/a	*Fallopia*	*Reynoutria*
**Haraldson (1978)**	Fallopiasect.Fallopia ; sect.Pleuropterus	*Muehlenbeckia*	Fallopiasect.Parogonum	*Reynoutria*; Fallopiasect.Pleuropterus
**Ronse Decraene and Akeroyd (1988)**	Fallopiasect.Fallopia ; sect.Sarmentosae	n/a	n/a	Fallopiasect.Reynoutria ; sect.Sarmentosae
**Bailey and Stace (1992)**	Fallopiasect.Fallopia ; sect.Sarmentosae	n/a	Fallopiasect.Parogonum	Fallopiasect.Reynoutria ; sect.Sarmentosae
**Brandbyge (1993)**	*Fallopia*	*Muehlenbeckia*	Fallopiasect.Parogonum	*Reynoutria*
**Galasso et al. (2009)**	*Fallopia*	*Muehlenbeckia*	n/a	*Reynoutria*
**Schuster et al. (2011b)**	*Fallopia*	*Muehlenbeckia*	n/a	*Reynoutria*
**Proposed classification**	Fallopiasect.Fallopia ; sect.Sarmentosae	*Muehlenbeckia*	*Parogonum*	*Reynoutria*

The latest molecular phylogenetic schemes, using plastid and nuclear sequence data, place *Reynoutria*, *Muehlenbeckia* and *Fallopia* in a strongly supported monophyletic group, known as the RMF clade (Schuster et al. 2011a, b, 2015). The stem age of this clade is reportedly 46.1–48.2 MYA (Schuster et al. 2013). Within this clade *Fallopia* and *Muehlenbeckia* are sister genera and appear to be more closely related to each other than either are to *Reynoutria*, which is immediately basal to them. *Coccoloba* and the rest of the Coccolobeae, previously regarded as members of subfamily Polygonoideae and sister to *Muehlenbeckia*, are now placed well away from it in subfamily Eriogonoideae (Cuénoud et al. 2002; Lamb Frye and Kron 2003; Galasso et al. 2009; Burke and Sanchez 2011).

### ﻿*Reynoutria*

*Reynoutria* is an East Asian genus (Ohwi 1965; Anjen and Park 2003b) and, as currently circumscribed by Schuster et al. (2011b), corresponds to Bentham and Hooker’s (1880)PolygonumsectPleuropterus, containing both the erect, strongly rhizomatous knotweeds (*R.japonica* s.l. and *R.sachalinensis*), as well as weakly rhizomatous climbers (*R.multiflora* (Thunb.) Moldenke and *R.ciliinervis* (Nakai) Moldenke). Within *Reynoutria* s.l., the erect, strongly rhizomatous knotweeds form an in-group, with *R.multiflora* as a basal lineage (Schuster et al. 2011a, b). This distinction between the erect and climbing taxa is further supported by examinations of secondary chemistry, which reveal two distinct chemical entities within the genus, both of which are separable from *Fallopia* s.s. (Kim et al. 2000b; Park et al. 2011). Indeed, Galasso et al. (2018) preferred to separate the two groups and retained the climbing taxa in the genus *Pleuropterus* Turcz.

*Reynoutria* was formerly amalgamated under *Fallopia* by Ronse Decraene and Akeroyd (1988), who argued that the anatomical heterogeneity within the two genera breaks down any clear distinction between them, particularly when the full range of taxa is taken into account. They instead emphasised similarities in stamen type, tepal vasculature and outer tepal morphology in support of merging the genera. Intergeneric hybrids also occur between *Reynoutria* and *Fallopia* (= × *Reyllopia* Holub) and have been taken to support amalgamation (Bailey 1988, 2001). In Ronse Decraene and Akeroyd’s (1988) treatment of *Fallopia*, the erect *Reynoutria* taxa are classified as Fallopiasect.Reynoutria (Houtt.) Ronse Decr., while *R.multiflora* is grouped with other perennial climbers (such as *F.baldschuanica* (Regel) Holub) in Fallopiasect.Sarmentosae (I.Grinț.) Holub. However, the latest phylogenetic schemes have shown that *Fallopia* sensu Ronse Decraene and Akeroyd (1988) is paraphyletic as species of *Muehlenbeckia* are nested within it (Galasso et al. 2009; Schuster et al. 2011a, b).

Species of *Reynoutria* are herbaceous, rhizomatous perennials with dry, winged mature perianths, paniculate inflorescences, fimbriate stigmas and are functionally gynodioecious or hermaphrodite (Haraldson 1978; Ronse Decraene and Akeroyd 1988). Chromosome base number is *x* = 11 (Bailey and Stace 1992; Kim and Park 2000).

*Reynoutriajaponica* s.l. is also comprised of a number of infraspecific and allied specific taxa. These include the tall, lowland form var.japonica (= *R.japonica* s.s.) and the dwarf, montane form var.compacta (= *R.compacta*), as well as East Asian endemics, such as var.uzenensis Honda (= *R.uzenensis* (Honda) Honda), var.terminalis (Honda) Kitag., *R.elliptica* (Koidz.) Migo ex Nakai and *R.forbesii* (Hance) T.Yamaz (Bailey 2003). var.uzenensis is a tall lowland form, characterised by pubescent foliage with uniseriate, multicellular hairs and occurs only in the north-eastern part of Honshu, Japan (Pashley 2003). var.terminalis is endemic to the Izu Islands, off the coast of Honshu and is characterised by large, lustrous leaves (Inamura et al. 2000). *Reynoutriaforbesii* is the name applied to knotweed growing on the Chinese mainland, which is sometimes treated as synonymous with *R.elliptica* from Korea. Both taxa have a distinctive elliptic leaf shape with a rounded base, as opposed to truncate like var.japonica and thick rigid hairs on the lower surface of the leaves (absent in var.japonica) (Anjen and Park 2003a; Bailey 2003; Galasso et al. 2009).

### ﻿*Fallopia*

*Fallopia* is a heterogeneous genus divided into three sections: sect. Fallopia, sect. Sarmentosae and sect. Parogonum Haraldson (Holub 1970; Haraldson 1978).

#### Fallopiasect.Fallopia

Fallopiasect.Fallopia was erected by Holub (1970) and contains approximately eight taxa: *F.convolvulus* (L.) Á.Löve, *F.cristata* (Engelm. & A.Gray) Holub, *F.dentatoalata* (F.Schmidt) Holub, *F.dumetorum* (L.) Holub, *F.filipes* (H.Hara) Holub, *F.pterocarpa* (Wall. ex Meisn.) Holub, *F.scandens* (L.) Holub (the type species) and *F.schischkinii* Tzvelev (Hara 1972; Tzvelev 1987; Kim et al. 2000c). Species of section Fallopia are annual vines with dry winged mature perianths (secondarily lost in *F.convolvulus* and *F.schischkinii*), spike-like to racemose inflorescences, capitate stigmas and perfect flowers (Haraldson 1978; Ronse Decraene and Akeroyd 1988). The section has a north temperate distribution (Hara 1982; Qaiser 2001; Anjen and Park 2003a; Freeman and Hinds 2005) and the chromosome base number is *x* = 10 (Bailey and Stace 1992).

An examination of secondary chemistry found that the flavonoid profiles of sect. Fallopia form a distinct group, which provides additional evidence for the segregation of sect. Fallopia within the genus (Kim et al. 2000a). Previous molecular phylogenetic studies also show that Fallopiasect.Fallopia forms a strongly supported monophyletic clade within the genus, which is sister to sect. Sarmentosae (Galasso et al. 2009; Schuster et al. 2011b).

#### Fallopiasect.Sarmentosae

Fallopiasect.Sarmentosae was erected by Holub (1970) and contains *F.aubertii* (L.Henry) Holub and *F.baldschuanica*, which may be conspecific (Bailey 1989; Bailey and Stace 1992). Species of sect. Sarmentosae are woody climbing perennials (without rhizomes) with dry winged mature perianths, paniculate inflorescences, capitate stigmas, and perfect flowers (Haraldson 1978; Ronse Decraene and Akeroyd 1988). The section has a central Asian distribution (Qaiser 2001; Anjen and Park 2003a) and the chromosome base number is *x* = 10 (Bailey and Stace 1992).

An examination of secondary chemistry found that the flavonoid profile of *F.baldschuanica* was distinct from other *Fallopia* species (Kim et al. 2000b). Previous molecular studies have also shown that Fallopiasect.Sarmentosae is a monophyletic group, sister to Fallopiasect.Fallopia (Galasso et al. 2009; Schuster et al. 2011b).

*Fallopiakoreana* B.U.Oh & J.G.Kim is a climbing perennial herb endemic to Korea. It is rhizomatous, has enlarged winged perianths in fruit that become twisted at the apex and capitate stigmas with projected surfaces (Oh and Kim 1996). Somatic chromosome number is reported as *2n* = 20 (Kim et al. 2000b). *Fallopiakoreana* was formerly classified in Fallopiasect.Pleuropterus (Kim et al. 2000b), but molecular work by Schuster et al. (2011b) indicates that it is sister to *F.baldschuanica* and may also belong in Fallopiasect.Sarmentosae. Due to a lack of available material, *F.koreana*, was not included in the present study.

#### Fallopiasect.Parogonum

Fallopiasect.Parogonum was erected by Haraldson (1978) and contains two taxa: *F.ciliinodis* (Michx.) Holub (the type species) and *F.cynanchoides* (Hemsl.) Haraldson. Species of sect. Parogonum are herbaceous perennial climbers, distinguished by their unique trichome type, a stiff unicellular hair with a papillate surface (Haraldson 1978; Bailey 1989). Members of the section also have dry mature perianths, which do not become enlarged and winged in fruit (cf. *F.convolvulus*), paniculate inflorescences, mildly-fimbriate stigmas and perfect flowers (Haraldson 1978). Section Parogonum has a disjunct East Asian-Eastern North American distribution with *F.ciliinodis* native to the East Coast of North America and *F.cynanchoides* restricted to Central China (Anjen and Park 2003a; Freeman and Hinds 2005). Chromosome base number is *x* = 11 (Bailey and Stace 1992; Kim et al. 2000a).

A molecular phylogenetic study including *F.ciliinodis* presented an unclear picture of its position within tribe Polygoneae (Schuster et al. 2015). Plastid data strongly supported its inclusion in the RMF clade (subtribe Reynoutriinae), but not within *Fallopia* s.s., while the inclusion of a nuclear dataset placed it outside of the RMF clade and weakly supported as sister to a DAP clade (subtribe Polygoninae), including: *Duma* T.M.Schust, *Atraphaxis* L. and Polygonums.s. The separation ofsect.Parogonum from *Fallopia* s.s. is supported by an examination of secondary chemistry, which found that the flavonoid profile of *F.ciliinodis* to be substantially different from the rest of *Fallopia*, most closely resembling the climbing *Reynoutria* taxa, *R.multiflora* and *R.ciliinervis* (Kim et al. 2000a, 2000b). However, species of sect. Parogonum were not included in Ronse Decraene and Akeroyd’s (1988) morphological treatment of the genus and *F.cynanchoides* has been missing from all molecular studies to date, so the placement of Fallopiasect.Parogonum within subtribe Reynoutriinae remained unclear until the present study.

### ﻿*Muehlenbeckia*

*Muehlenbeckia* was erected by Meisner (1840) to include five species of *Polygonum* with *M.australis* (G.Forst.) Meisn. as the type. The genus, as currently understood, contains approximately twenty-seven species, eighteen from Australia, New Zealand and the Pacific Islands and nine from Central and South America (Schuster et al. 2013).

The taxa of *Muehlenbeckia* are variable in habit, ranging from prostrate, mat-forming creepers to erect shrubs to woody lianas; all are perennial and none is herbaceous. *Muehlenbeckia* species have succulent mature perianths, as opposed to dry and winged as in *Fallopia* and *Reynoutria*, fasciculate to racemose to paniculate inflorescences, fimbriate stigmas and are often dioecious (Allan 1961; Brandbyge 1992; Green et al. 1994). Chromosome base number is *x* = 10 (Beuzenberg and Hair 1983; de Lange and Murray 2002).

Meisner (1856) divided *Muehlenbeckia* into three sections, namely sect. Sarcogonum Endl., sect. *Eumühlenbeckia* Endl. and sect. Andinia Wedd., based upon floral characters. The latest molecular phylogenetic schemes (e.g. Schuster et al. (2011a, b, 2013, 2015)) have revealed that *Muehlenbeckia* contains three well-supported subclades, denoted *x*, *y* and *z*, which generally correspond with biogeographic distribution and bear little resemblance to Meisner’s (1856) sectional treatment. Clade *x* is a predominantly New Zealand clade, containing: *M.complexa* (A.Cunn.) Meisn., *M.ephedroides* Hook.f. and *M.axillaris* (Hook.f.) Endl., as well as *M.tuggeranong* Mallinson, an Australian endemic (Makinson and Mallinson 1997). Clade *y* is an Australian clade, containing: *M.arnhemica* K.L.Wilson & R.O.Makinson, *M.diclina* (F.Muell) F.Muell., *M.rhyticarya* F.Muell. ex Benth. and *M.zippelii* (Meisn.) Danser with strong support, as well as *M.adpressa* (Labill.) Meisn., *M.gracillima* Meisn., *M.costata* K.L.Wilson & R.O.Makinson and *M.gunnii* (Hook.f.) Endl. with weaker support. Clade *z* is a predominantly Central/South American clade, containing: *M.urubambensis* Brandbyge, *M.volcanica* (Benth.) Endl., *M.tiliifolia* Wedd., *M.tamnifolia* (Kunth) Meisn. and, somewhat surprisingly, *M.australis*, a native of New Zealand and Norfolk Island, whose inclusion in this clade was hypothesised to be the result of long-distance dispersal (Schuster et al. 2013). The phylogenetic placements of two further species are unresolved by previous analyses: *M.astonii* Petrie, a divaricating shrub native to New Zealand and *M.platyclada* (F.Muell.) Meisn. (= *Homalocladiumplatycladum* (F.Muell.) L.H.Bailey), an evergreen shrub with phylloclades, native to New Guinea and the Solomon Islands (e.g. Schuster et al. (2011a,b)).

In the current study, we further resolved the evolutionary relationships of *Reynoutria*, *Fallopia* and *Muehlenbeckia* within subtribe Reynoutriinae by including the widest sampling of ingroup taxa for the clade to date, in particular being the first to include infraspecific taxa and allies of *R.japonica*, as well as both taxa of Fallopiasect.Parogonum. A phylogenetic analysis was conducted on sequence data from six markers: two nuclear, the second intron of *LEAFY* (*LEAFYi2*) and the internal transcribed spacer (ITS) of the 17S-5.8S-26S rDNA region; and four plastid, *matK*, *rbcL*, *rps16-trnK* and *trnL-trnF*.

## ﻿Materials and methods

### ﻿Plant material

An accession list for the current study is presented in Suppl. material 2. Samples were collected either as fresh material or taken from herbarium specimens with the curator’s permission. Where possible, voucher specimens were made and deposited in the University of Leicester Herbarium (**LTR**).

The accessions, collected for the current study, represent the widest sampling of in-group taxa for any phylogenetic study in this subtribe to date (cf. Galasso et al. (2009); Schuster et al. (2011b, 2015)). In total, nine *Reynoutria*, nineteen *Muehlenbeckia* and nine *Fallopia* taxa were included. Published taxa that are missing from the current study include: *F.filipes*, *F.koreana*, *F.pterocarpa*, *F.schischkinii*, *M.andina* Brandbyge, *M.fruticulosa* (Walp.) Standl., *M.hastulata* (Sm.) I.M.Johnst., *M.monticola* Pulle, *M.nummularia* H.Gross, *M.polybotrya* Meisn., *M.sagittifolia* (Ortega) Meisn. and *M.triloba* Danser.

### ﻿Molecular analysis

#### DNA extraction, amplification and sequencing

Total genomic DNA was isolated from dried leaf material using the DNeasy Plant Mini Kit (Qiagen). Six markers, four plastid: *matK*, *rbcL*, *rps16-trnK* and *trnL-trnF* and two nuclear: ITS and *LEAFYi2*, were amplified by PCR. The primer sequences and cycling conditions are presented in Suppl. material 3. For the ITS, the reaction mixture was supplemented with 4% DMSO to prevent amplification of paralogous pseudogenes (Buckler et al. 1997). PCR amplicons were visualised by gel electrophoresis, purified using the NucleoSpin Gel and PCR Clean-up kit (Machery-Nagel) and Sanger-sequenced by GATC Biotech (Konstanz, Germany). *LEAFYi2* was also sequenced from clones. Cloning was conducted using the pGEM-T Easy Vector System (Promega) and α-Select Competent Cells taken from E. coli (Bioline). Recombinant plasmids were selected by blue-white screening and the size of the insert determined by colony PCR with M13 primers. Plasmid DNA was isolated from cell cultures using the E.Z.N.A. Plasmid Mini Kit (Omega Bio-tek) and a minimum of five colonies were sequenced per accession.

### ﻿Alignment and phylogenetic analysis

Generated sequence reads were viewed, trimmed and edited with Geneious R7 (created by Biomatters; available from http://www.geneious.com/). The sequences were then blasted against the NCBI GenBank database to ensure taxon and gene matches. In total, 259 sequences were used, 107 (41%) of these were newly generated for the current study and 152 (59%) were downloaded from the NCBI GenBank database (Suppl. material 1).

Multiple sequences were aligned for each gene region using the Clustal W algorithm (Larkin et al. 2007). Indels and areas of ambiguous homology were excised from the alignments prior to phylogenetic analysis. The collective chloroplast dataset (*matK*, *rbcL*, *rps16-trnK*, *trnL-trnF*), *LEAFYi2* and the ITS were analysed separately and then concatenated to produce a total evidence dataset. Not all gene regions were available for all taxa and some taxa had incomplete datasets (Table 2; Suppl. material 1). Missing data were treated as a continuous series of Ns in concatenated datasets (Wiens 2006).

**Table 2.** Statistical values for analysed datasets.

Dataset	Aligned length (bp)	No. (%) conserved characters	No. (%) variable characters	No. (%) parsimony informative characters	No. (%) of missing species
*matK*	1224	893 (73)	331 (27)	188 (15)	2 (4)
*rbcL*	1327	1140 (86)	182 (14)	105 (8)	15 (28)
*rps16-trnK*	1034	729 (71)	305 (29)	137 (13)	20 (37)
*trnL-trnF*	935	643 (69)	292 (31)	155 (16)	6 (11)
ITS	767	465 (61)	302 (39)	199 (26)	3 (6)
*LEAFYi2*	930	541 (58)	389 (42)	174 (19)	22 (40)
cp combined	4510	3403 (76)	1107 (24)	584 (13)	0 (0)
Total combined	6207	4420 (71)	1787 (29)	951 (15)	–

Two methods were used to infer the evolutionary relationships of the taxa from the datasets, Maximum Likelihood (ML) and Maximum Parsimony (MP). ML analysis was conducted using PhyML 3.0 (Guindon and Gascuel 2003). The most appropriate model of DNA sequence evolution for each dataset was estimated using Model Selection in MEGA6 (Tamura et al. 2013) and the model with the lowest Bayesian information criterion chosen. Topology searches for the most likely tree were carried out using the nearest-neighbour interchange (NNI) search strategy. Maximum Parsimony (MP) analysis was conducted using PAUP* 4.0 (Swofford 2002). Topology searches for the most parsimonious trees were carried out using a branch and bound search strategy with the addition method FURTHEST. Node support for ML and MP analyses was estimated by resampling inferred trees by bootstrapping (BS) - 1000 replicates (Felsenstein 1985). Two species of *Coccoloba* were selected to form the outgroup as they belong to the sister subfamily Eriogonoideae and their separation from in-group taxa is well established (Cuénoud et al. 2002; Lamb Frye and Kron 2003; Sanchez et al. 2009, 2011; Burke and Sanchez 2011). They could also be reliably aligned with in-group taxa for the all markers, excluding *LEAFYi2*. Phylogenetic trees were generated for individual nuclear (*LEAFYi2*, ITS), combined chloroplast (*matK*, *rbcL*, *rps16-trnK* and *trnL-trnF*) and total evidence (*LEAFYi2*, the ITS, *matK*, *rbcL*, *rps16-trnK* and *trnL-trnF*) datasets. Congruence between trees was determined by comparison of BS values.

### ﻿Data availability statement

All sequences generated for this study have been deposited on GenBank (NCBI). Sequence alignments are available in the Suppl. materials 4–7.

## ﻿Results

**Figure 1.**
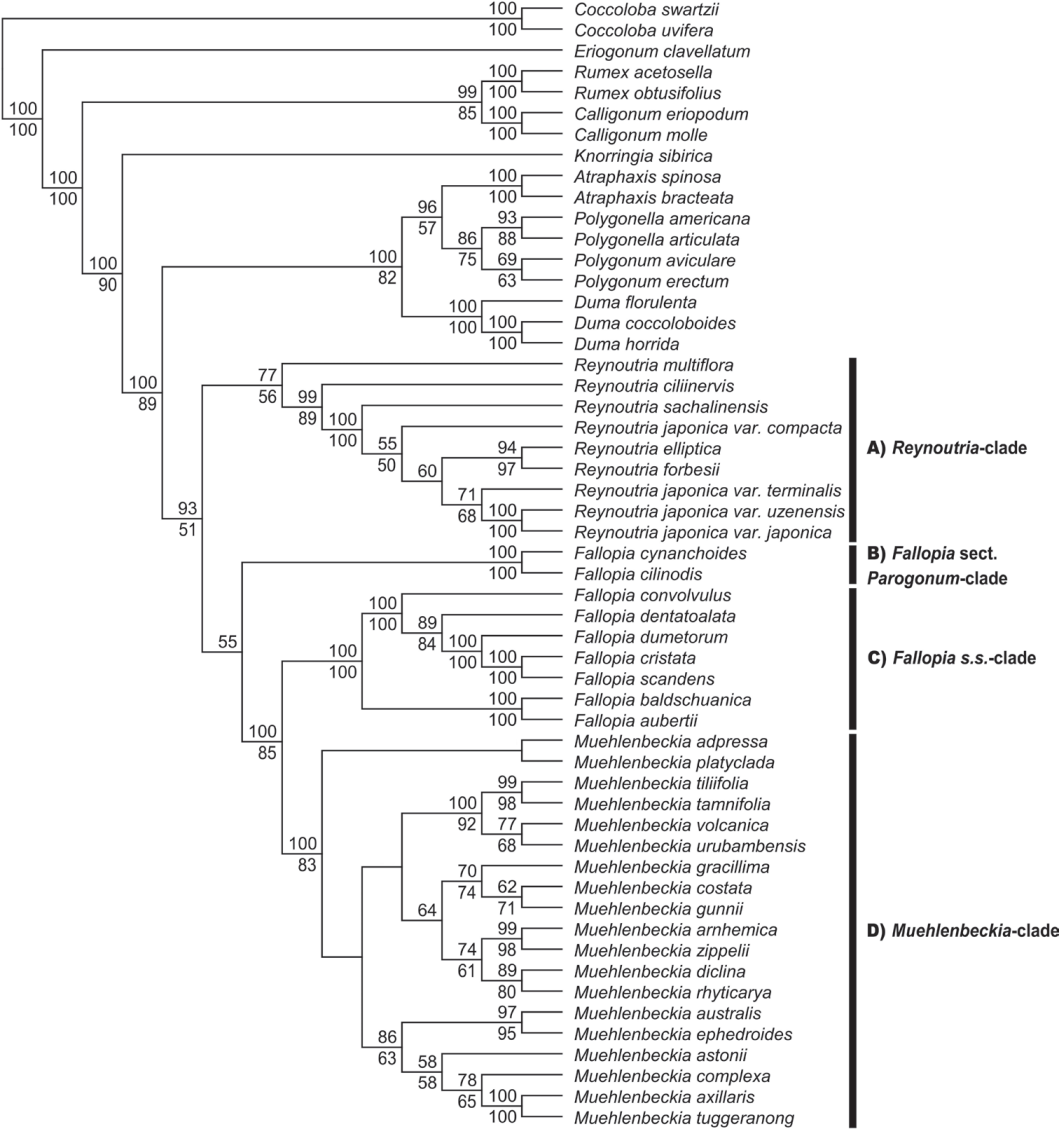
A total evidence phylogenetic tree generated by a Maximum Likelihood analysis of concatenated nuclear (ITS and *LEAFYi2*) and chloroplast (*matK*, *rbcL*, *rps16-trnK* and *trnL-trnF*) sequence data. Bootstrap support values (≥ 50%) are displayed above and below the nodes for Maximum Likelihood and Maximum Parsimony analyses, respectively. Maximum Parsimony analysis recovered eight equally parsimonious trees (3099 steps). The main clades within subtribe Reynoutriinae are marked with bars.

Phylogenetic trees were generated by ML and MP. The two analyses were largely congruent, although bootstrap support (BS) values for ML were generally higher. The trees presented (Fig. 1 and Suppl. material 1: figs S1–S3) follow the topology generated by ML analysis. BS values (≥ 50%) are displayed above and below branches for ML and MP, respectively. Hyphens (-) indicate nodes where MP trees differ from ML in branching order. BS values from ML analysis are cited in the main text, unless otherwise stated.

The ITS, *LEAFYi2* and combined chloroplast (*matK*, *rbcL*, *rps16-trnK* and *trnL-trnF*) datasets were analysed separately (Suppl. material 1: figs S1–S3). The single-marker analyses of nuclear loci (the ITS and *LEAFYi2*) produced poorly-resolved trees and branches with strong support were largely confined to termini (Suppl. material 1: figs S1, S2), while the combined chloroplast analysis was more informative with good resolution at internal nodes (Suppl. material 1: fig. S3). The three independent datasets (two nuclear and chloroplast) were largely congruent and so were concatenated to form a total evidence tree (Fig. 1). The total evidence tree agreed with the combined chloroplast tree, excepting the placement of R.japonicavar.compacta and *M.ephedroides*: 1) In the combined chloroplast tree, R.japonicavar.compacta was sister to *R.sachalinensis* with strong support (84% BS), while in the total evidence tree, it was placed in a weakly-supported *R.japonica* s.l. clade (55%), to which *R.sachalinensis* was basal; 2) In the combined chloroplast analyses *M.ephedroides* fell within a clade with *M.axillaris* and *M.tuggeranong* (78% BS), while in the total evidence analysis, it was sister to *M.australis* (97% BS).

The results of the total evidence analysis gave greater resolution and a higher number of strongly-supported nodes than the individual nuclear and combined chloroplast trees alone. In our view, the total evidence tree is the best estimate of the phylogenetic relationships in this study; hereafter, this is the tree described (unless otherwise stated) and forms the basis of our discussions.

### ﻿Phylogenetic analysis

Subtribe Reynoutriinae formed a well-supported (93% BS) clade within the Polygonaceae; sister to a subtribe Polygoninae clade (100% BS). Within subtribe Reynoutriinae, there were four well-supported subclades: A) a *Reynoutria* clade (77% BS with *R.multiflora* and 99% BS without); B) a Fallopiasect.Parogonum clade (100% BS); C) a *Fallopia* s.s. clade (100% BS) and D) a *Muehlenbeckia* clade (100% BS). The *Fallopia* s.s. and *Muehlenbeckia* clades were sister to one another with the Fallopiasect.Parogonum clade immediately basal to them (100% BS) and the *Reynoutria* clade basal to all three (55% BS).

The *Reynoutria* clade contained *R.multiflora*, *R.ciliinervis*, *R.sachalinensis*, R.japonicavar.compacta, *R.elliptica*, *R.forbesii*, R.japonicavar.terminalis, R.japonicavar.uzenensis and R.japonicavar.japonica. Within the clade, the erect *Reynoutria* taxa formed a strongly-supported subclade (100% BS), with *R.ciliinervis* (99% BS) and *R.multiflora* (77% BS) as independent basal lineages. However, relationships within the erect subclade were poorly resolved with only weakly-supported internal nodes. Nevertheless, there were two clear subclades within it, a strongly-supported one containing *R.elliptica* + *R.forbesii* (94% BS) and a moderately-supported one containing R.japonicavar.terminalis, R.japonicavar.japonica + R.japonicavar.uzenensis (71% BS).

The Fallopiasect.Parogonum clade contained the sister taxa *F.cynanchoides* and *F.ciliinodis* with strong support (100% BS). The *Fallopia* s.s. clade contained two strongly-supported subclades, C1) a sect. Fallopia clade (100% BS) and C2) a sect. Sarmentosae clade (100% BS). The sect. Fallopia clade contained *F.convolvulus*, *F.dentatoalata*, *F.dumetorum*, *F.cristata* and *F.scandens*. All relationships within the clade there were strongly supported. Within this clade, *F.cristata* and *F.scandens* were almost identical (> 99.85% pairwise identity for all available sequence data) and were placed as sister taxa (100% BS). The sect. Sarmentosae clade contained the sister taxa *F.baldschuanica* and *F.aubertii* with strong support (100% BS).

The *Muehlenbeckia* clade contained three subclades with moderate to strong support, although the relationships between them were entirely unresolved: D1) a Central/South American clade (100% BS); D2) an Australian clade (64% BS) and D3) a predominantly New Zealand clade (86% BS). The American clade contained *M.tiliifolia*, *M.tamnifolia*, *M.volcanica* and *M.urubambensis*. All relationships within the clade were strongly supported. Within this clade, there were two pairs of sister taxa, *M.tiliifolia* + *M.tamnifolia* (99% BS) and *M.volcanica* + *M.urubambensis* (77% BS). The Australian clade contained *M.gracillima*, *M.costata*, *M.gunnii*, *M.arnhemica*, *M.zippelii*, *M.diclina* and *M.rhyticarya*. All relationships within the clade were moderately/strongly supported. Within this clade, there were two subclades, one containing *M.gracillima*, *M.costata* and *M.gunnii* (70% BS) and another containing *M.zippelii*, *M.arnhemica*, *M.diclina* and *M.rhyticarya* (74% BS). The predominantly New Zealand clade contained *M.australis*, *M.ephedroides*, *M.astonii*, *M.complexa*, *M.axillaris*, as well as the Australian endemic *M.tuggeranong*. All relationships within the clade were moderately/strongly supported. In this clade, there were two subclades, one containing *M.australis* + *M.ephedroides* (97% BS) and another containing *M.astonii*, *M.complexa*, *M.axillaris* + *M.tuggeranong* (58% BS). Within this second subclade, *M.axillaris* and *M.tuggeranong* were sister taxa with strong support (100% BS). The placement of *M.platyclada* and *M.adpressa* within the genus was unresolved.

### ﻿*LEAFYi2* copy number

*LEAFYi2* was single-copy in all diploid taxa and was sequenced directly, but in two polyploid taxa, R.japonicavar.japonica and *F.convolvulus*, two amplicons of different size were observed and these were sequenced from clones (Suppl. material 1: fig. S2). In R.japonicavar.japonica, the two copies were sister to one another (73% BS), while in *F.convolvulus*, the two copies were separate on the tree. Copy 1 was sister to *F.dumetorum* (100% BS), while the position of copy 2 was unresolved in the ML analysis (< 50% BS), but placed in a clade with *F.scandens* and *F.cristata* in the MP analysis (70% BS).

## ﻿Discussion

### ﻿Phylogenetic relationships

The species of Reynoutriinae form a strongly-supported monophyletic clade within the Polygonaceae. This clade is characterised by the presence of extra-floral nectaries at the base of leaf petioles (Salisbury 1909; Brandbyge 1992; Schuster et al. 2011b) and *Tiniaria* pollen type (Hedberg 1946; Brandbyge 1992). The subtribe has a cosmopolitan distribution and is found in the Northern and Southern Hemispheres (Allan 1961; Brandbyge 1992; Anjen and Park 2003a, 2003b; Freeman and Hinds 2005). Within the Reynoutriinae clade, there are four strongly-supported subclades: a *Reynoutria* clade, a Fallopiasect.Parogonum clade, a *Fallopia* s.s. clade (containing Fallopiasect.Fallopia and sect. Sarmentosae) and a *Muehlenbeckia* clade. *Fallopia* s.s. and *Muehlenbeckia* are sister to one another, while Fallopiasect.Parogonum is basal to them and *Reynoutria* is basal to all three.

### ﻿*Reynoutria* clade

*Reynoutria* taxa form a strongly-supported monophyletic clade within subtribe Reynoutriinae, which confirms the findings of previous molecular studies (e.g. Galasso et al. (2009); Schuster et al. (2011a, b, 2015)). The clade has an East Asian distribution and is characterised by the presence of rhizomes, which are unique within the subtribe (Ohwi 1965; Anjen and Park 2003a, b).

Within the *Reynoutria* clade, the erect taxa form a strongly-supported subclade (100% BS). Indeed, previous authors (e.g. Galasso et al. (2018)) have considered the erect taxa as distinct from the climbing taxa (*R.multiflora* & *R.ciliinervis*) and retain the climbers in their own genus, *Pleuropterus*. However, this is not supported by the current study as *R.multiflora* and *R.ciliinervis* do not form a reciprocally monophyletic subclade, but rather they form separate basal lineages within the *Reynoutria* clade. We, therefore, continue to treat both the climbing and erect taxa as *Reynoutria* s.l. (in line with Schuster et al. (2011b)), until further evidence is accumulated.

Within the erect *Reynoutria* clade, *R.japonica* and its allies form a weakly-supported monophyletic subclade, with *R.sachalinensis* as sister. Within this subclade, the notorious invasive alien var.japonica is most closely related to the other tall lowland forms from Japan, var.uzenensis and var.terminalis, which most likely represent subspecies of *R.japonica*.

*Reynoutriaforbesii* from China and *R.elliptica* from Korea are sister taxa and form a monophyletic group, which comes out as sister to *R.japonica* with weak support. Furthermore, *R.forbesii* and *R.elliptica* are very similar morphologically and they most likely represent a single taxon - the epithet *forbesii* is the older name has priority (Anjen and Park 2003a; Bailey 2003; Galasso et al. 2009). Whether *R.forbesii* is specifically distinct from *R.japonica* remains unclear and further analysis using a wider range of material from across the native range is required. In the interim, we continue to treat this taxon as *R.forbesii*, with *R.elliptica* as a synonym.

The placement of the high-altitude dwarf form R.japonicavar.compacta differed between the individual nuclear and combined chloroplast analyses, being sister to *R.sachalinensis* on the chloroplast tree (as also demonstrated by Galasso et al. (2009)) and closer to *R.japonica* on the nuclear trees. This is most likely due to reticulate evolution with the chloroplast haplotype of *R.sachalinensis* being captured during the formation of R.japonicavar.compacta ; Var.compacta is also distinct in being of small stature and flowering earlier, as well as having undulate leaf margins, somewhat leathery leaves and a red-tinged inflorescence (Ohwi 1965; Desjardins et al. 2022), characteristics which are maintained even when transplanted at lower altitudes (Shiosaka and Shibata 1993). This morphological distinction, its montane habitat and reticulate history can all be taken to support species status as *R.compacta* (Galasso et al. 2009). However, the distinction between the tall lowland forms of *R.japonica* and dwarf montane *compacta*, while apparent in the small subset of adventive clones, is less clear in the native range where leaf morphology and height grade into one another along an altitudinal cline (Bailey 2003). As is the case with *R.forbesii*, further analysis using a wider sampling of material from the native range is required to determine the true taxonomic status of var.compacta and whether it should be treated as a species in its own right or a subspecies of *R.japonica*.

### ﻿Fallopiasect.Parogonum clade

*Fallopiaciliinodis* and *F.cynanchoides* form a strongly-supported monophyletic clade within subtribe Reynoutriinae, characterised by papillate trichomes (Haraldson 1978; Bailey 1989). *Fallopiacynanchoides* is restricted to central China (Anjen and Park 2003a) and *F.ciliinodis* to the East Coast of North America (Freeman and Hinds 2005). Fallopiasect.Parogonum, therefore, represents a good example of a well-known floristic affinity, in which counterparts (conspecifics or intercontinental species pairs) are discontinuously distributed between East Asia and Eastern North America (Graham 1972). This disjunct distribution is the product of complex processes, including migration/dispersal, extinction, speciation and vicariance, but the general pattern is thought to be due to the exchange of taxa between Eurasia and North America over the Bering and North Atlantic land bridges in the mid-Tertiary, followed by extirpation in western North America and North East Asia in the cooling climates of the late Tertiary to early Quaternary (Wen 1999, 2001).

The position of Fallopiasect.Parogonum within subtribe Reynoutriinae has been the subject of some speculation. Schuster et al. (2011b) predicted that the species of Fallopiasect.Parogonum may belong to the *Reynoutria* clade due to perceived similarities in morphology, for example, paniculate inflorescences, multicellular trichomes, chromosome base number (*x* = 11; Bailey and Stace (1992)) and secondary chemistry (Kim et al. 2000a, b). However, in the current study, Fallopiasect.Parogonum appeared to be more closely related to *Muehlenbeckia* and the rest of *Fallopia* than to *Reynoutria*. This placement was strongly supported by the combined chloroplast analysis, but only weakly supported by the total evidence analysis.

### ﻿*Fallopia* s.s. clade

The species of Fallopia sect. Parogonum, formed a strongly-supported monophyletic clade within subtribe Reynoutriinae and are characterised within the subtribe by capitate stigmas. Within the *Fallopia* s.s. clade, there are two strongly-supported subclades, corresponding to Fallopiasect.Fallopia and Fallopiasect.Sarmentosae, which are sister to one another.

The species of sect. Fallopia form a strongly-supported subclade within the *Fallopias.s.* clade, which confirms the results of previous molecular studies (Galasso et al. 2009; Schuster et al. 2011b) and supports Holub’s (1970) treatment of them as a separate section. Members of this subclade are characterised by their annual twining habits, few-flowered inflorescences and distinctive flavonoid profiles (Kim et al. 2000a). All members of this subclade are found in the north temperate region (Anjen and Park 2003a; Freeman and Hinds 2005; Stace 2019).

The analysis also indicated that *F.cristata* is not specifically distinct from *F.scandens*. The phylogenetic analysis placed them as sister to one another and they were almost identical for the markers analysed. The two taxa are thought to be separable on the basis of their mature perianths, which are said to be smaller and more narrowly winged in *F.cristata* (Freeman and Hinds 2005). However, these differences are only apparent in extreme specimens and intermediate forms are often encountered that gradually grade into *F.scandens* (Freeman and Hinds 2005). Furthermore, morphometric (Kim et al. 2000c) and chemotaxonomic (Kim et al. 2000a) studies suggest that *F.cristata* falls within the normal variability of *F.scandens*. In our view, it is not worthy of taxonomic recognition.

The species of sect. Sarmentosae form a strongly-supported subclade within the *Fallopia* s.s. clade, which supports Holub’s (1970) treatment of them as a section within the genus. Members of this subclade can be identified by a combination of characters: capitate stigmas, dry mature perianths and a woody perennial habit. They also have distinctive flavonoid profiles (Kim et al. 2000b) and are native to Asia (Qaiser 2001; Anjen and Park 2003a).

### ﻿*Muehlenbeckia* clade

The species of *Muehlenbeckia* sampled formed a strongly-supported monophyletic clade within subtribe Reynoutriinae, which confirms the results of Schuster et al. (2011a, b) and supports Meisner’s (1840, 1856) treatment of them as a distinct group. Members of this clade are characterised by their succulent mature perianths and are found exclusively in the Southern Hemisphere (Allan 1961; Brandbyge 1992; Green et al. 1994). Within *Muehlenbeckia*, evolutionary relationships generally correspond to geographic distribution and there are three subgroups, a Central/South American clade, an Australian clade and a predominantly New Zealand clade.

The placement of *Muehlenbeckia* taxa in the current study is largely congruent with that of Schuster et al. (2011b) and bootstrap values are roughly equivalent. However, there is disagreement in the positions of two taxa. In Schuster et al. (2011b), *M.australis*, a native to New Zealand and Norfolk Island, is placed within the Central/South American clade with strong support, while in the current study, it falls, as one would more naturally expect, in the predominantly New Zealand clade. We have not seen the specimen used by Schuster et al. (2011b) (W.R. Barker 8995 & R.M. Barker; AD), but we are confident in the identity of the *M.australis* sample included in the current study. It was collected from Ōtari-Wilton’s Bush, Wellington, New Zealand by Dr Peter de Lange (Unitec Institute of Technology, New Zealand) and is supported by seven further collections of *M.australis* from around New Zealand, which form a monophyletic group within the New Zealand clade (Schmid et al., unpublished). Schuster et al.’s (2011b) analyses also failed to resolve the position of *M.astonii* within *Muehlenbeckia*, while, in the current analysis *M.astonii* was placed in the predominantly New Zealand clade with strong support. An examination of the sequence data used by Schuster et al. (2011b) revealed that the ITS sequence (EF635479) is likely a pseudogene, which inflated sequence divergence and resulted in the artificial separation of *M.astonii* from the rest of New Zealand *Muehlenbeckia*. This pseudogene was identified by its relatively low GC content (60.1% versus 65.5%) and the high number of SNPs in the conserved 5.8S region (Buckler et al. 1997; Álvaraez and Wendel 2003; Feliner and Rosselló 2007). We found that pseudogenised ITS copies would readily amplify in this group if 4% DMSO, or some other denaturant, was omitted from the PCR mixture.

The placement of *M.ephedroides* was incongruent between the chloroplast and individual nuclear analyses. In the chloroplast analyses, *M.ephedroides* fell within a clade with *M.axillaris* and *M.tuggeranong*, while in the nuclear analyses, it was sister to *M.australis*. As is the case in R.japonicavar.compacta, *M.ephedroides* likely has a reticulate history and, during its formation, appears to have captured the haplotype of an ancestor of *M.axillaris*/*M.tuggeranong*. This scenario is supported by observations of modern hybridisation in New Zealand *Muehlenbeckia* (Yong 1967).

### ﻿Taxonomy of Reynoutriinae

Reynoutriinae, or the RMF clade, is monophyletic and contains three genera *Reynoutria*, *Muehlenbeckia* and *Fallopia*. However, as currently circumscribed, *Fallopia* is paraphyletic as *Muehlenbeckia* is nested between Fallopiasect.Parogonum and the rest of the genus. The subtribe, therefore, requires an immediate taxonomic revision. There are two possible systematic interpretations to restore monophyly in this group, either treat *Fallopia*, *Muehlenbeckia* and *Reynoutria* as a single genus, *Fallopia*, which has priority, or treat Fallopiasect.Parogonum as a genus in its own right.

Both an amalgamated and a divided *Fallopia* can be supported by the available molecular data and there are putative synapomorphies for both treatments. An amalgamated *Fallopia* would include all members of the RMF clade and would be characterised by the presence of extra-floral pit nectaries at the base of leaf petioles and the *Tiniaria* pollen type (Salisbury 1909; Hedberg 1946; Brandbyge 1992), while a divided subtribe Reynoutriinae would be split into the different subclades of the RMF clade and these would be characterised by a number of putative synapomorphies: *Fallopia* by its capitate stigmas, *Parogonum* by its papillate trichomes, *Reynoutria* by its rhizomes and *Muehlenbeckia* by its succulent mature perianth (Brandbyge 1992; Anjen and Park 2003a, b; Freeman and Hinds 2005).

The two alternative treatments of the subtribe are both perfectly tenable and there are arguments for and against amalgamation. The arguments for amalgamating the genera are threefold: 1) The morphological characters used to separate *Fallopia*, *Muehlenbeckia* and *Reynoutria* are rather inconsistent. Meisner (1840, 1856) considered species of *Muehlenbeckia* distinct on the basis of their succulent mature perianths, fimbriate stigmas and dioecious breeding systems. However, fimbriate stigmas and functional dioecy are also found in *Reynoutria*. The only character that seems to consistently separate *Muehlenbeckia* is its succulent mature perianth (Brandbyge 1993), but, as Haraldson (1978) argues, succulent mature perianths have evolved several times within the Polygonaceae, for example, *Coccoloba*, *Duma*, *Muehlenbeckia* and *Persicaria* Mill. and is not a reliable character when delimiting genera. Schuster et al. (2011b) also cited basic chromosome basic number as a means of distinguishing *Reynoutria* (*x* = 11) from *Muehlenbeckia* and *Fallopia* (*x* = 10). However, the inclusion of Fallopiasect.Parogonum (*x* = 11) in a clade with *Muehlenbeckia* and *Fallopia* s.s. breaks down this distinction. Furthermore, intrageneric variation in basic chromosome number is not uncommon in the Polygonaceae, for example, *Persicaria*, *x* = 10, 11, 12 (Kim et al. 2008); 2) There are good synapomorphies for an amalgamated *Fallopia*, such as the presence of extra-floral pit nectaries and the *Tiniaria* pollen type (Salisbury 1909; Hedberg 1946; Brandbyge 1992); 3) Hybridisation occurs between the subclades, *Reynoutria* × *Fallopia* and *Reynoutria* × *Muehlenbeckia* (Bailey 2001, 2013).

Meanwhile, the arguments against amalgamating the genera are fivefold: 1) *Muehlenbeckia* has been treated as a distinct entity since its formation, while *Fallopia* and *Reynoutria* have often been treated as separate genera (Galasso et al. 2009); 2) It would require more taxonomic upheaval to amalgamate *Muehlenbeckia* within *Fallopias.l* and a greater number of name changes; 3) *Muehlenbeckia* is a well-established genus and in widespread usage amongst botanists in the Southern Hemisphere; 4) *Muehlenbeckia* has been conserved against previous priority challenges (Rickett and Stafleu 1959); 5) *Muehlenbeckia* has a distinct biogeographical distribution, being confined to the Southern Hemisphere and is clearly separate from northern *Fallopia* and *Reynoutria*.

On balance, we are of the opinion that, despite compelling arguments in favour of amalgamation, species of subtribe Reynoutriinae are better treated as multiple genera to limit nomenclatural upheaval, preserve names in widespread use and to better distinguish the clades. Fallopiasect.Parogonum has, therefore, been treated as a genus in its own right and the relevant binomial changes have been made below.

### ﻿Putative allopolyploid origin of *F.convolvulus*

*Fallopiaconvolvulus* (Fallopiasect.Fallopia) is tetraploid (2*n* = 40), but it is not known if it arose by autopolyploidy or allopolyploidy (Bailey and Stace 1992). In the current study, two divergent copies of the single-copy nuclear gene *LEAFYi2* were detected in *F.convolvulus*, which were clearly separated on the phylogenetic tree. One copy was sister to Eurasian *F.dumetorum*, while the other appeared to be most closely related to American *F.cristata*/*F.scandens*. The presence of two divergent copies can be taken as evidence for an allopolyploid origin of *F.convolvulus*, which may have originated as a result of hybridisation between the ancestors of *F.dumetorum* (2*n* = 20) and *F.scandens*/*F.cristata* (2*n* = 20), followed by chromosomal doubling. Bailey (1989) conjectured that *F.convolvulus* is derived from *F.scandens* and diversified relatively recently to become a weed of cereal crops. An allopolyploid origin of *F.convolvulus* is in line with this, as it would provide a mechanism for reproductive isolation and near-instantaneous speciation. Indeed, modern hybrids between *F.convolvulus* and *F.dumetorum*, *F.×convolvuloides* (Brügger) Holub, are triploid and sterile (Holub 1970).

However, this conclusion is not wholly supported by the other available datasets. In the combined chloroplast analysis *F.convolvulus* was placed basal to the rest of sect. Fallopia and was not sister to *F.dumetorum* or *F.scandens*/*F.cristata*. Furthermore, in the ITS analysis, only one functional copy was detected in *F.convolvulus*, but this is not unexpected given the homogenising processes of concerted evolution in tandemly-arranged repetitive DNA, such as the ITS (Álvarez and Wendel 2003). A genomic in situ hybridisation (GISH) experiment using labelled *F.dumetorum* and *F.scandens* genomes to probe *F.convolvulus* chromosomes would be highly informative.

## ﻿Conclusion

Subtribe Reynoutriinae is a monophyletic group, which is characterised by the presence of extra-floral, nectariferous glands at the base of leaf petioles. Within the subtribe, four main clades were identified, which represent separate genera: East Asian *Reynoutria*, disjunct East Asian/Eastern North American *Parogonum* (Haraldson) Desjardins & J.P.Bailey, gen. et stat. nov., north temperate *Fallopia* and austral *Muehlenbeckia*. Within the subtribe, *Reynoutria* can be identified by the presence of rhizomes, *Parogonum* by stiff papillate hairs, *Fallopia* by capitate stigmas and *Muehlenbeckia* by succulent mature perianths.

### ﻿Nomenclatural novelties

#### ﻿ Parogonum

Taxon classificationPlantaeCaryophyllalesPolygonaceae

(Haraldson) Desjardins & J.P.Bailey, gen. et stat. nov.

E31836CE-ABAA-556A-8412-68D814CB5217

urn:lsid:ipni.org:names:77315139-1

Fallopia sect. Parogonum Haraldson, *Symb. Bot. Upsal.* 22: 78 (1978). Basionym.

#### ﻿1) Parogonum ciliinode

Taxon classificationPlantaeCaryophyllalesPolygonaceae

(Michx.) Desjardins & J.P.Bailey comb. nov.

F541B2F7-1C2D-531B-9F3E-4248EE7C6ED0

urn:lsid:ipni.org:names:77315140-1

Polygonum ciliinode (‘*cilinode*’) Michx., *Fl. Bor.-Amer.* (Michaux) 1: 241 (1803). Basionym.
Tiniaria ciliinodis (‘*cilinodis*’) (Michx.) Small, *Fl. S.E. U.S. [Small].*: 382 (1903). Bilderdykiaciliinodis (‘*cilinodis*’) (Michx.) Greene, *Leafl. Bot. Observ. Crit.* 1: 23 (1904). Reynoutriaciliinodis (‘*cilinodis*’) (Michx.) Shinners, Sida 3: 117 (1967). Fallopiaciliinodis (‘*cilinodis*’) (Michx.) Holub, *Folia Geobot. Phytotax.* 6: 176 (1970). Homotypic synonyms.

#### ﻿2) Parogonum cynanchoides

Taxon classificationPlantaeCaryophyllalesPolygonaceae

(Hemsl.) Desjardins & J.P.Bailey comb. nov.

0BA1DE33-6D0E-525B-BD1D-DA4ED82539F0

urn:lsid:ipni.org:names:77315141-1

Polygonum cynanchoides Hemsl., *J. Linn. Soc.*, *Bot.* 26: 338 (1891). Basionym.
Fallopia cynanchoides (Hemsl.) Haraldson, *Symb. Bot. Upsal.* 22: 78 (1978). Homotypic synonym.

#### ﻿3) Parogonum cynanchoides subsp. glabriusculum

Taxon classificationPlantaeCaryophyllalesPolygonaceae

(A.J.Li) Desjardins & J.P.Bailey, comb. et stat. nov.

583A4EAB-4D58-54D6-8DE5-7D616C7DBD41

urn:lsid:ipni.org:names:77315142-1

Polygonum cynanchoides var. glabriusculum A.J.Li, *F. Xizang* 1: 608 (1983). Basionym.
Fallopia cynanchoides var. glabriuscula (A.J.Li) A.J.Li, *Fl. Reipubl. Popularis Sin.* 25: 104 (1998). Homotypic synonym.

## Supplementary Material

XML Treatment for Parogonum

XML Treatment for Parogonum ciliinode

XML Treatment for Parogonum cynanchoides

XML Treatment for Parogonum cynanchoides subsp. glabriusculum

## Supplementary materials

Supplementary material 1 Additional phylogenetic trees from ITS, *LEAFYi2* and combined chloroplast analyses. Data type pdf file Explanation note **fig S1.** A phylogenetic tree generated by a Maximum Likelihood analysis of ITS sequence data. Bootstrap support values (≥ 50%) are displayed above and below the nodes for Maximum Likelihood and Maximum Parsimony analyses, respectively. Maximum Parsimony analysis recovered two equally parsimonious trees (825 steps); **fig S2.** A phylogenetic tree generated by a Maximum Likelihood analysis of *LEAFYi2* sequence data. Bootstrap support values (≥5 0%) are displayed above and below the nodes for Maximum Likelihood and Maximum Parsimony analyses, respectively. Hyphens (-) indicate nodes where parsimony and likelihood trees differ in branching pattern. Maximum Parsimony analysis recovered three equally parsimonious trees (598 steps); **fig S3.** A phylogenetic tree generated by a Maximum Likelihood analysis of concatenated chloroplast sequence data (*matK*, *rbcL*, *trnL-trnF* & *rps16-trnK*). Bootstrap support values (≥ 50%) are displayed above and below the nodes for Maximum Likelihood and Maximum Parsimony analyses, respectively. Maximum Parsimony analysis recovered 191 equally parsimonious trees (1600 steps). The main clades within subtribe Reynoutriinae are marked with bars. https://binary.pensoft.net/file/816594This dataset is made available under the Open Database License (http://opendatacommons.org/licenses/odbl/1.0/). The Open Database License (ODbL) is a license agreement intended to allow users to freely share, modify, and use this Dataset while maintaining this same freedom for others, provided that the original source and author(s) are credited.Stuart D. Desjardins, John P. Bailey, Baowei Zhang, Kai Zhao, Trude Schwarzacher

Supplementary material 2 Accessions used in the current study Data type excel file https://binary.pensoft.net/file/816595This dataset is made available under the Open Database License (http://opendatacommons.org/licenses/odbl/1.0/). The Open Database License (ODbL) is a license agreement intended to allow users to freely share, modify, and use this Dataset while maintaining this same freedom for others, provided that the original source and author(s) are credited.Stuart D. Desjardins, John P. Bailey, Baowei Zhang, Kai Zhao, Trude Schwarzacher

Supplementary material 3 Primer sequences and PCR cycling conditions Data type excel file https://binary.pensoft.net/file/816596This dataset is made available under the Open Database License (http://opendatacommons.org/licenses/odbl/1.0/). The Open Database License (ODbL) is a license agreement intended to allow users to freely share, modify, and use this Dataset while maintaining this same freedom for others, provided that the original source and author(s) are credited.Stuart D. Desjardins, John P. Bailey, Baowei Zhang, Kai Zhao, Trude Schwarzacher

Supplementary material 4 ITS multiple-sequence alignment Data type .txt file https://binary.pensoft.net/file/816597This dataset is made available under the Open Database License (http://opendatacommons.org/licenses/odbl/1.0/). The Open Database License (ODbL) is a license agreement intended to allow users to freely share, modify, and use this Dataset while maintaining this same freedom for others, provided that the original source and author(s) are credited.Stuart D. Desjardins, John P. Bailey, Baowei Zhang, Kai Zhao, Trude Schwarzacher

Supplementary material 5 *LEAFYi2* multiple-sequence alignment Data type .txt file https://binary.pensoft.net/file/816598This dataset is made available under the Open Database License (http://opendatacommons.org/licenses/odbl/1.0/). The Open Database License (ODbL) is a license agreement intended to allow users to freely share, modify, and use this Dataset while maintaining this same freedom for others, provided that the original source and author(s) are credited.Stuart D. Desjardins, John P. Bailey, Baowei Zhang, Kai Zhao, Trude Schwarzacher

Supplementary material 6 Combined chloroplast multiple-sequence alignment Data type .txt file https://binary.pensoft.net/file/816599This dataset is made available under the Open Database License (http://opendatacommons.org/licenses/odbl/1.0/). The Open Database License (ODbL) is a license agreement intended to allow users to freely share, modify, and use this Dataset while maintaining this same freedom for others, provided that the original source and author(s) are credited.Stuart D. Desjardins, John P. Bailey, Baowei Zhang, Kai Zhao, Trude Schwarzacher

Supplementary material 7 Total evidence multiple-sequence alignment Data type .txt file https://binary.pensoft.net/file/816600This dataset is made available under the Open Database License (http://opendatacommons.org/licenses/odbl/1.0/). The Open Database License (ODbL) is a license agreement intended to allow users to freely share, modify, and use this Dataset while maintaining this same freedom for others, provided that the original source and author(s) are credited.Stuart D. Desjardins, John P. Bailey, Baowei Zhang, Kai Zhao, Trude Schwarzacher
