# Supplementary material for: ﻿New insights into the phylogenetic relationships of Japanese knotweed (Reynoutriajaponica) and allied taxa in subtribe Reynoutriinae (Polygonaceae)
Source: PhytoKeys. 2023 Feb 27;220:83–108. doi: 10.3897/phytokeys.220.96922 (PMC10209619; doi:10.3897/phytokeys.220.96922)
Supplement: Supplementary material 1 — Additional phylogenetic trees from ITS, LEAFYi2 and combined chloroplast analyses. [file phytokeys-220-083_article-96922__-s001.pdf]

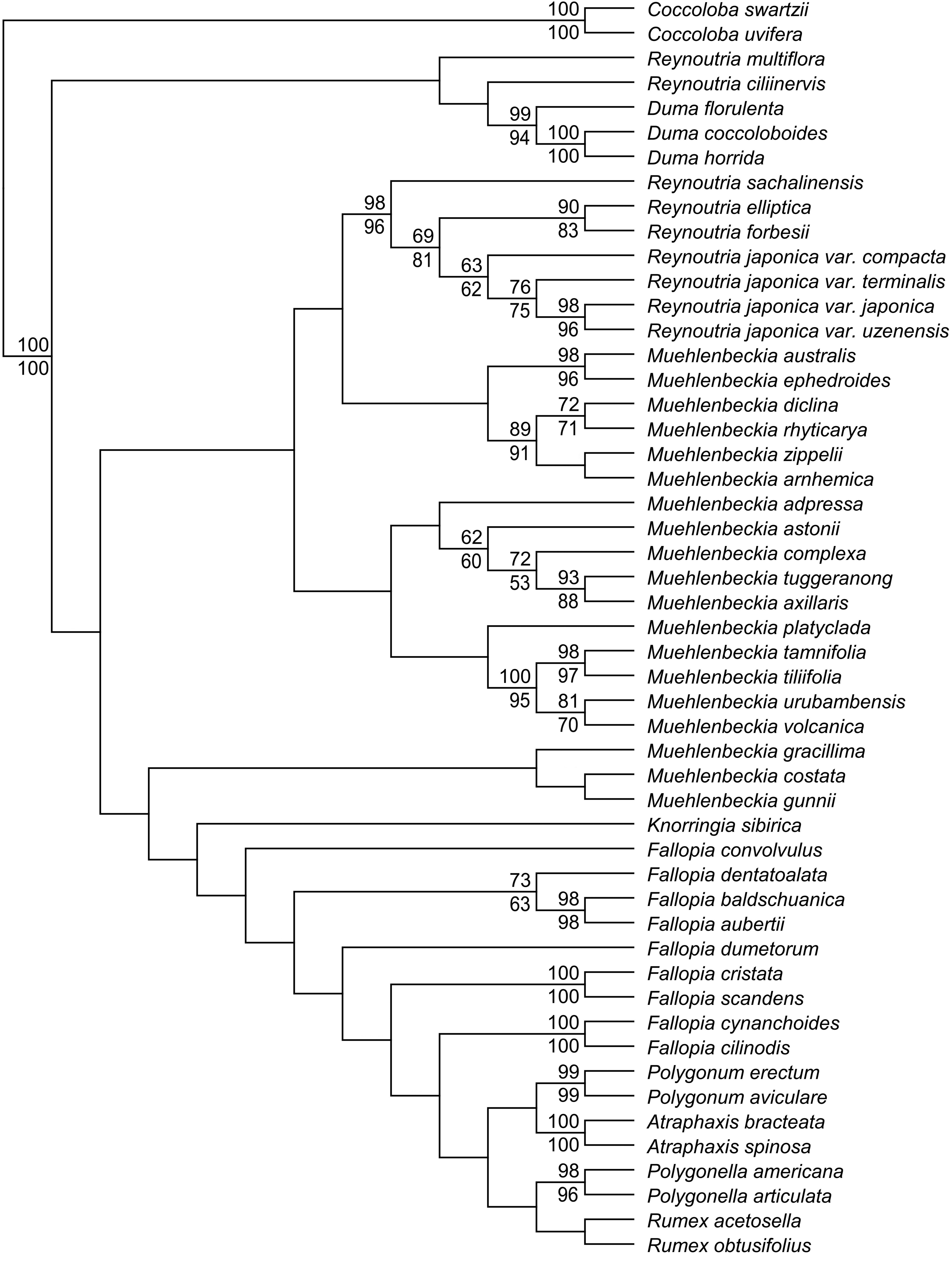

SFigure 1. A phylogenetic tree generated by a Maximum Likelihood analysis of ITS sequence data. Bootstrap support values (≥ 50%) are displayed above and below the nodes for Maximum Likelihood and Maximum Parsimony analyses, respectively. Maximum Parsimony analysis recovered two equally parsimonious trees (825 steps).

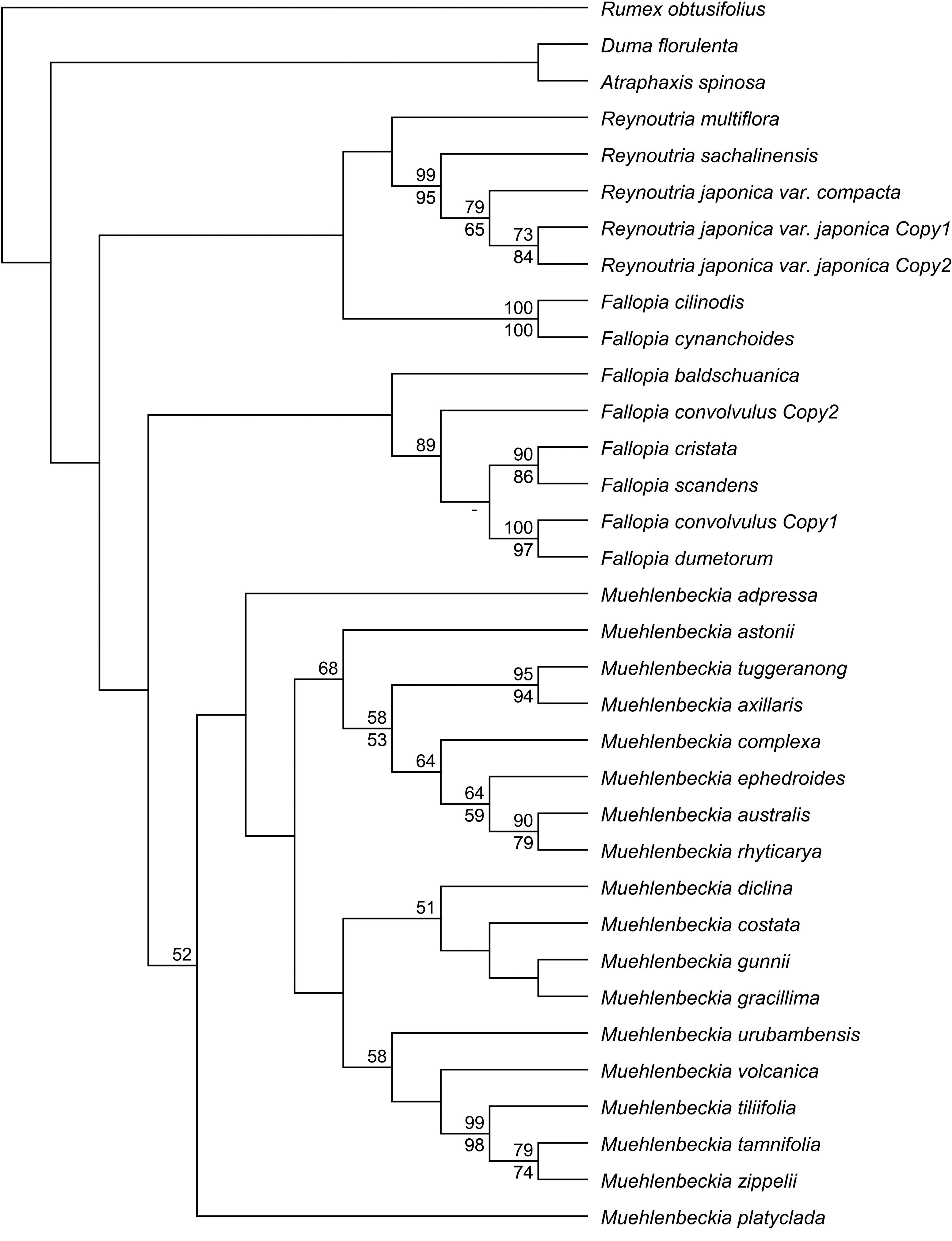

SFigure 2. A phylogenetic tree generated by a Maximum Likelihood analysis of *LEAFYi2* sequence data. Bootstrap support values (≥5 0%) are displayed above and below the nodes for Maximum Likelihood and Maximum Parsimony analyses, respectively. Hyphens (-) indicate nodes where parsimony and likelihood trees differ in branching pattern. Maximum Parsimony analysis recovered three equally parsimonious trees (598 steps).

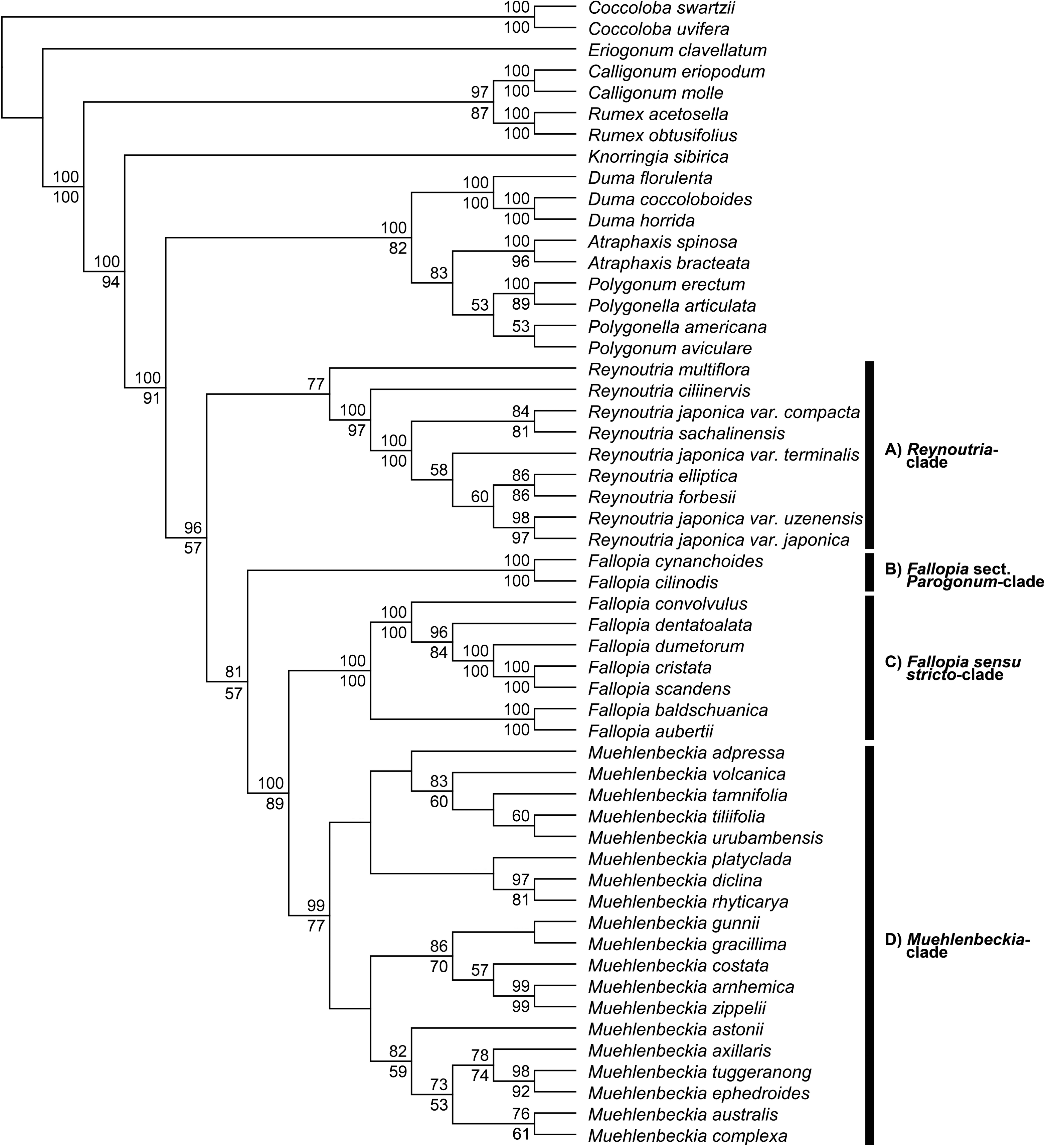

SFigure 3. A phylogenetic tree generated by a Maximum Likelihood analysis of concatenated chloroplast sequence data (*matK, rbcL, trnL-trnF* & *rps16-trnK*). Bootstrap support values (≥ 50%) are displayed above and below the nodes for Maximum Likelihood and Maximum Parsimony analyses, respectively. Maximum Parsimony analysis recovered 191 equally parsimonious trees (1600 steps). The main clades within subtribe Reynoutriinae are marked with bars.
